# Alterations in the gut virome in patients with ankylosing spondylitis

**DOI:** 10.3389/fimmu.2023.1154380

**Published:** 2023-03-30

**Authors:** Chen Li, Yan Zhang, Qiulong Yan, Ruochun Guo, Changming Chen, Shenghui Li, Yue Zhang, Jinxin Meng, Jie Ma, Wei You, Zhisong Wu, Wen Sun

**Affiliations:** ^1^ Department of Rheumatology, Fangshan Hospital, Beijing University of Chinese Medicine, Beijing, China; ^2^ Department of Traditional Chinese Medicine, Beijing Friendship Hospital, Capital Medical University, Beijing, China; ^3^ Department of Microbiology, College of Basic Medical Sciences, Dalian Medical University, Dalian, China; ^4^ Puensum Genetech Institute, Wuhan, China; ^5^ Department of Rheumatology and Immunology, The Second Affiliated Hospital of Guizhou University of Traditional Chinese Medicine, Guiyang, China; ^6^ School of Traditional Chinese Medicine, Beijing University of Chinese Medicine, Beijing, China; ^7^ Beijing Key Laboratory of Acupuncture Neuromodulation, Department of Acupuncture and Moxibustion, Beijing Hospital of Traditional Chinese Medicine, Capital Medical University, Beijing, China; ^8^ Department of Intensive Care Medicine, Dongfang Hospital Beijing University of Chinese Medicine, Beijing, China; ^9^ Key Laboratory of Health Cultivation of the Ministry of Education, Beijing University of Chinese Medicine, Beijing, China; ^10^ Beijing Key Laboratory of Health Cultivation, Beijing University of Chinese Medicine, Beijing, China

**Keywords:** ankylosing spondylitis, gut virome, viral dysbiosis, viral operational taxonomic units, viral function, metagenome sequencing

## Abstract

**Introduction:**

Ankylosing spondylitis (AS), a chronic autoimmune disease, has been linked to the gut bacteriome.

**Methods:**

To investigate the characteristics of the gut virome in AS, we profiled the gut viral community of 193 AS patients and 59 healthy subjects based on a metagenome-wide analysis of fecal metagenomes from two publicly available datasets.

**Results:**

AS patients revealed a significant decrease in gut viral richness and a considerable alteration of the overall viral structure. At the family level, AS patients had an increased abundance of *Gratiaviridae* and *Quimbyviridae* and a decreased abundance of *Drexlerviridae* and *Schitoviridae*. We identified 1,004 differentially abundant viral operational taxonomic units (vOTUs) between patients and controls, including a higher proportion of AS-enriched *Myoviridae* viruses and control-enriched *Siphoviridae* viruses. Moreover, the AS-enriched vOTUs were more likely to infect bacteria such as *Flavonifractor*, *Achromobacter*, and *Eggerthellaceae*, whereas the control-enriched vOTUs were more likely to be *Blautia*, *Ruminococcus*, *Collinsella*, *Prevotella*, and *Faecalibacterium* bacteriophages. Additionally, some viral functional orthologs differed significantly in frequency between the AS-enriched and control-enriched vOTUs, suggesting the functional role of these AS-associated viruses. Moreover, we trained classification models based on gut viral signatures to discriminate AS patients from healthy controls, with an optimal area under the receiver operator characteristic curve (AUC) up to 0.936, suggesting the clinical potential of the gut virome for diagnosing AS.

**Discussion:**

This work provides novel insight into the AS gut virome, and the findings may guide future mechanistic and therapeutic studies for other autoimmune diseases.

## Introduction

Ankylosing spondylitis (AS) is a chronic autoimmune disease that mainly affects the sacroiliac joints and the spine; ankylosis and spinal deformity can occur in severe cases (1). The prevalence rate of AS in mainland China is approximately 0.29%, and the ratio of males to females is approximately 2~3:1. The disease often occurs in people aged 20~30 years old and is rare in those over 40 years and under 8 years old (2). AS can lead to physiological injury and disability (3), resulting in loss of physical function, decreased quality of life, and heavy financial burden on patients, families, and society (4).

Current studies suggest that AS is a genetic disease that is closely associated with HLA-B27 (5). The pathogenesis of AS includes host genetic factors and environmental triggers, such as the gut microbiota (6). Increasing evidence also linked AS to some gastrointestinal disorders, such as irritable bowel syndrome (IBS) and inflammatory bowel disease (IBD) (7, 8). AS and IBD share similarities in terms of genetic risk factors and pathogenesis, and interaction with the gut microbiome is closely related to the pathogenesis of IBD (7). Therefore, AS may also be a gut microbiome-driven disease. The gut microbiome is considered to play an important role in the human defense system, with a wide range of physiological roles. Disturbance of interactions between the immune system and the gut microbiome may induce complex pathological changes (9). Clinical studies have shown that up to 70% of patients with AS have subclinical intestinal inflammation, and 5~10% of these patients will develop IBD, which is the common extra-articular manifestation of AS (10). In addition, a study of 211 Chinese subjects showed that AS was characterized by severe gut dysbiosis, increased relative abundances of *Prevotella melaninogenica*, *Prevotella copri*, and *Prevotella* sp. *C561*, and decreased *Bacteroides* spp (11). Another study showed that the microbiota characteristics of AS patients included increased *Proteobacteria* and decreased *Bacteroidetes* abundances, which was due to enrichment of *Escherichia-Shigella*, *Veillonella, Lachnospiraceae* NK4A136 group and reduction of *Prevotella* strain 9, *Megamonas*, and *Fusobacterium* (12). These studies suggest that the gut microbiota is associated with AS and may even be one of the critical inducing factors of AS. As the gut microbiota contains multiple components, including bacteria, fungi, and viruses, an obvious gap in knowledge is the role each component plays.

Although studies to date have focused on gut bacteria as one of the main components of the human gut, gut viruses also deserve attention. The human gut viral community is mainly composed of bacteriophages, most of which remain undiscovered, and very little is known about their role in the formation of the gut microbiome and its impact on human health. The virome is closely related to the gut microbiota, and recent studies have highlighted the association between the gut virome and many diseases, especially IBD (13), autoimmune diseases (14), and type 2 diabetes mellitus (15). Furthermore, evidence that the virome directly affects human health through the immune system has been found (16, 17). More importantly, our previous study has preliminary showed significant changes in the gut virome associated with several immune diseases, including AS (18). Given these findings, it makes sense to investigate the association between AS and the virome in more detail.

Overall, some viruses with low abundance or that are difficult to sequence might not be detected through metagenomic sequencing technology. However, gut virome studies can be performed by virus-like particle (VLP) metagenomic sequencing (19–21). By combining metagenomic and VLP metagenomic sequencing analyses, researchers can obtain a highly comprehensive map of the gut virus population. In this study, to identify changes in the gut virome in AS, we reanalyzed raw data from publicly available metagenomic data for two cohorts, which included a total of 252 human fecal samples (193 AS patients and 59 healthy subjects). Specifically, we compared the viral composition of AS patients with that of healthy individuals based on metagenome-based sequencing data and investigated the relationship between viruses and bacteria. In light of the exploration of the gut virome of AS patients, a better understanding of the etiology and pathogenesis of AS will contribute to developing prevention and treatment strategies for AS from new perspectives.

## Methods

### Sample information

This study involved 252 fecal metagenomic sequencing samples from two public cohorts (**Table S1**). The first cohort (cohort A) was recruited from Guangzhou, South China, and comprised 113 patients with AS and 37 healthy controls (HCs) (22). The other cohort (cohort B) included 80 AS patients and 22 HCs living in Beijing, North China, after removing 40 HC samples from another study to avoid batch effects (23). The sample metadata of cohort B is available in Zhou et al. study. For cohort B, AS patients were treatment-naive individuals, and had no significant difference in age (Student’s t test, p value = 0.87) and body mass index (Student’s t test, p value = 0.63) compared with HCs.

### Preprocessing of metagenomic datasets

The raw metagenomic datasets were downloaded from the National Center for Biotechnology Information (NCBI) Sequence Read Archive (SRA) database with project accession IDs PRJEB28545 and PRJEB29373, respectively. The downloaded raw reads were filtered using fastp v0.20.1 (24) with the options ‘-l 90 -q 20 -u 30 -y –trim_poly_g’ to remove low-quality reads. The filtered reads were mapped to the GRCh38 reference genome using bowtie2 v2.4.1 (25) with default options to remove human reads. Finally, we obtained an average of 8.98 Gb clean data per sample (after quality control and human read removal).

### Gut viral reference

To analyze the composition of the gut viral community in metagenomic samples, we used a gut virus catalog comprising over 67,000 nonredundant viral operational taxonomic units (vOTUs) as the reference. This Chinese gut virus catalog (cnGVC) was constructed from over 10,000 publicly available fecal metagenomes from the Chinese population (including all samples used in the current study) (18). Briefly, the clean reads of each metagenomic sample were assembled into contigs using Megahit v1.2.9 (26). Only contigs ≥5 kb in length were used to identify viral sequences in each sample. Identification, decontamination, and dereplication of viral sequences were performed according to our prior study (27). The quality of viral sequences was estimated using CheckV v0.7.0 (28). Taxonomic classification, host prediction, and functional annotation of viral sequences were also performed according to our prior study (18).

### Taxonomic profiling of the gut virome

To generate taxonomic profiles of gut viral communities, the clean reads in each metagenomic sample were mapped to cnGVC using bowtie2 v2.4.1 with options ‘–end-to-end –fast –no-unal’. The total mapping reads of all samples were randomly subsampled to the same sequencing amount (1.2 million). The relative abundance of each vOTU was measured as the ratio of the number of reads mapped to the vOTU to the total number of reads mapped to any vOTU in each metagenome. In addition, the relative abundance of each viral family was generated by summing the relative abundances of vOTUs annotated with the corresponding family.

### Statistical analyses

Statistical analysis and visualization were implemented in R v4.0.3 (29).

#### Alpha and beta diversities

Gut virome diversity was estimated based on the profiles at the vOTU level. The observed number of vOTUs was measured as the number of vOTUs with the relative abundance of >0. Shannon and Simpson diversity indices were measured by the *diversity* function in the *vegan* package. Bray-Curtis distances between samples were measured by the *vegdist* function in the *vegan* package. Principal coordinate analysis (PCoA) of Bray-Curtis distances was carried out *via* the *pcoa* function in the *ape* package. Permutational multivariate analysis of variance (PERMANOVA) was carried out using the *adonis* function in the *vegan* package.

#### Identification of viral markers

First, the identification of viral markers at the vOTU-level was performed between AS patients and healthy controls in each cohort using the *wilcox.test* function. We further performed Fisher’s method for combining p-values from independent tests in two cohorts by the *sumlog* function in the *metap* package. The combined p-values were adjusted *via* the *p.adjust* function with the option ‘method=BH’. A vOTU with adjusted p-value (FDR) of < 0.05 was recognized as a AS-associated viral marker.

#### Comparison analysis of functions between AS- and HC-enriched vOTUs

The occurrence rate of each KO in a group was measured as the number of vOTUs with this KO divided by the total number of vOTUs in the group. Fisher’s exact test was performed using the *fisher.test* function to determine whether the occurrence rate of each KO differed significantly between two groups. Then the obtained p-values were adjusted *via* the *p.adjust* function with the option ‘method=BH’. A KO with adjusted p-value (FDR) of < 0.05 was deemed to be significantly different between two groups.

#### Classification models

The intra-cohort models based on AS-associated viral markers were constructed using the *randomForest* function followed by 5 times of 5 fivefold cross-validations, and their performances were evaluated based on AUC scores calculated by the *roc* function in the *pROC* package. The cross-dataset model was evaluated by training it on one cohort and testing it on the other. The importance ranking of viral markers was obtained by the *importance* function.

## Results

### Study population and gut virome composition

**Figure 1:**
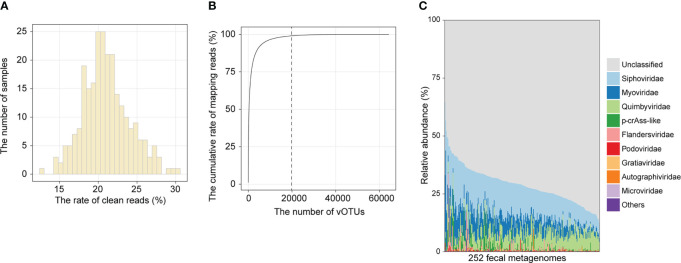
Overview of the gut viral community. **(A)** Histogram showing the proportion of metagenomic reads mapped to cnGVC. **(B)** The accumulative rate of mapping reads as a function of the number of vOTUs. A total of 67,096 vOTUs were sorted in descending order based on their average relative abundance across all metagenomic samples. **(C)** The gut viral composition of all samples at the family level.

To establish the composition of the gut viral community, we mapped clean reads of each sample to the cnGVC database, including 67,096 dereplicated vOTUs with high completeness (18). The rates of reads mapping to cnGVC ranged from 12.68% to 30.24% (**Figure 1A**), suggesting efficient capture of gut viral reads. Notably, 19,844 of 67,096 vOTUs accounted for 99% of mapping reads across all samples (**Figure 1B**) and were further used to characterize the gut viral composition of the two cohorts. Similar to previous studies (30, 31), the dominant families in the gut viral composition included *Siphoviridae* (mean relative abundance, 13.7%), *Myoviridae* (6.4%), *Quimbyviridae* (6.1%), and *crAss-like* viruses (1.4%), in addition to unclassified viruses (**Figure 1C**).

### Gut viral structure and diversity in ankylosing spondylitis

**Figure 2:**
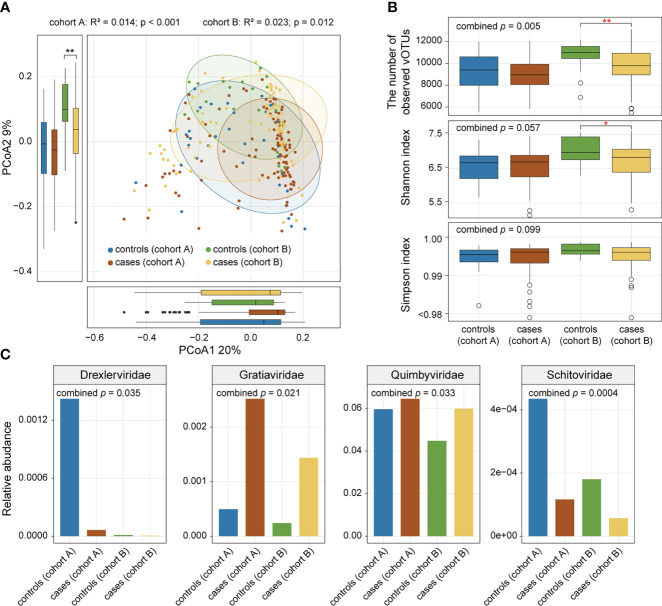
Comparison of gut viral diversity and composition between AS patients and healthy controls. **(A)** PCoA of Bray-Curtis distances of the gut virome at the vOTU level. Samples are shown at the first and second principal coordinates (PCoA1 and PCoA2), and the ratio of variance contributed by these two PCoAs is shown. Ellipsoids represent a 95% confidence interval surrounding each group. The bottom and left boxplots show the sample scores in PCoA1 and PCoA2 (boxes show medians/quartiles; error bars extend to the most extreme values within 1.5 interquartile ranges). **(B)** Boxplot showing the number of observed vOTUs (upper panel), Shannon diversity index (middle panel), and Simpson diversity index (bottom panel) of the gut virome of all samples. Wilcoxon rank-sum test: *p<0.05; **p<0.01. **(C)** Bar plot showing relative abundances of differentially abundant viral families between patients and controls.

We performed PCoA based on the Bray-Curtis distance of the vOTU-level profiles to assess the difference in gut virome composition between AS patients and healthy controls and visualized the first two principal coordinate axes explaining 29% of the total variation (**Figure 2A**). The first principal coordinates of AS samples were larger than those of HC samples, and the direction of the effect of disease status was consistent across the two cohorts. Similarly, the gut viral composition of the two cohorts showed a relationship with disease status in the same direction along the second principal coordinate. In addition, PERMANOVA showed a significant difference in the overall virome composition between AS patients and HCs within each cohort (**Figure 2A**; *adonis*, p value <0.05).

To estimate the effect of disease status on gut viral richness and evenness, alpha diversities of each sample were calculated using three indices (i.e., number of observed vOTUs, Shannon diversity index, and Simpson index). Comparison analysis was conducted based on Fisher’s method of combining p values from Wilcoxon rank-sum tests. AS patients showed a significant decrease in viral richness (number of observed vOTUs) compared with controls (combined p value = 0.005), particularly in cohort A (Wilcoxon rank-sum test, p value = 0.002; **Figure 2B**). However, there was no significant difference in viral evenness (Shannon index and Simpson index) between the two groups (combined p value > 0.05; **Figure 2B**).

Comparison of the family-level composition revealed that AS patients had an increased abundance of *Gratiaviridae* and *Quimbyviridae* but that HCs had a higher abundance of *Drexlerviridae* and *Schitoviridae* (combined p value < 0.05; **Figure 2C**) in their viromes.

### Identification of viral signatures associated with ankylosing spondylitis

**Figure 3:**
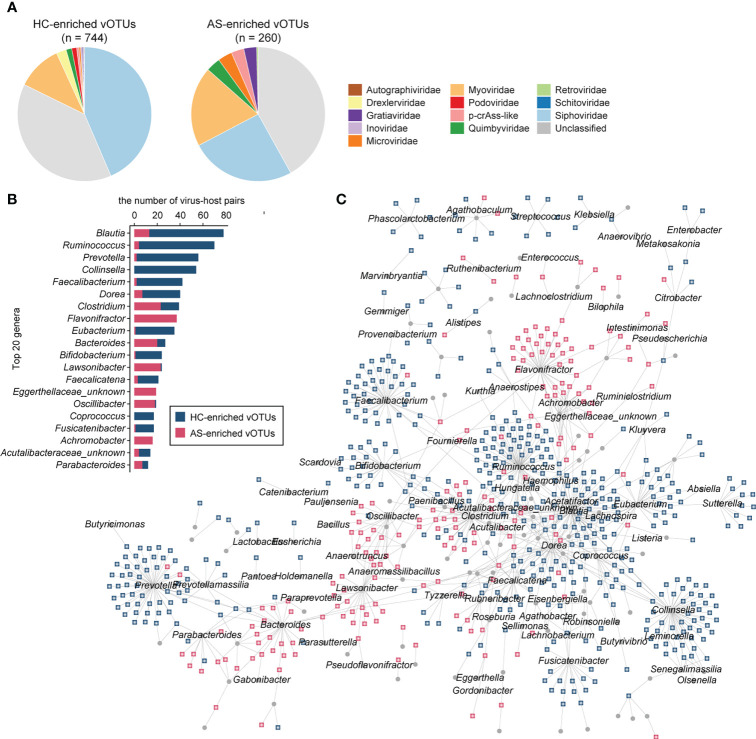
AS-associated gut viral signatures. **(A)** Taxonomic distribution of AS-enriched and HC-enriched vOTUs. **(B)** Distribution of prokaryotic hosts of AS-associated vOTUs. **(C)** Network showing vOTUs and corresponding prokaryotic hosts.

We obtained 1,004 vOTUs with a significant difference between AS patients and HCs using the combined probability values from independent tests in two cohorts (BH-adjusted combined p value < 0.05), with 260 of these enriched in AS patients and 744 enriched in HCs (**Table S2**). For both the AS- and HC-enriched vOTUs, differential vOTUs were dominated by viruses belonging to *Siphoviridae*, *Myoviridae*, and unclassified taxa (**Figure 3A**). However, AS-enriched vOTUs presented a clearly higher proportion of *Myoviridae* viruses (19.2% vs. 10.9%); HC-enriched vOTUs contained more *Siphoviridae* viruses (43.5% vs. 25.4%). Moreover, AS-enriched vOTUs included more viruses belonging to *Microviridae* (9 vs. 3), *p-crAss-like* (8 vs. 4), and *Gratiaviridae* (8 vs. 2), whereas 18 *Drexlerviridae* and 9 *Podoviridae* viruses were observed among HC-enriched vOTUs, but no member of these two families was regarded as AS-enriched vOTUs.

Then, we shaped the virus-host infection networks based on the alignment of genomic homology and the matching of CRISPR spacer sequences according to a previous method (27, 32) and observed a remarkable difference in host preference between AS-enriched and HC-enriched vOTUs (**Figure 3B**). Most AS-associated vOTUs (66.2%, n = 665) could be assigned to at least one prokaryotic host, including 474 HC-enriched vOTUs and 191 AS-enriched vOTUs. The HC-enriched vOTUs are predicted to more frequently infect members of *Blautia* (such as *B. wexlerae* and *B. obeum*)*, Ruminococcus* (such as *R. callidus* and *R. bicirculans*), *Collinsella* (such as *C. aerofaciens*), *Prevotella*, *Faecalibacterium* (such as *F. prausnitzii*), *Dorea* (such as *D. longicatena and D. formicigenerans*), *Eubacterium* (such as *E. hallii and E. ventriosum*), and *Bifidobacterium* (such as *B. adolescentis*, *B. breve*, *and B. catenulatum*) (**Figure 3C**). In contrast to the HC-enriched vOTUs, the dominant hosts of the AS-enriched vOTUs included members of *Flavonifractor*, *Lawsonibacter*, *Clostridium*, *Bacteroides*, *Oscillibacter*, *Achromobacter* and an unknown genus of Eggerthellaceae. Strikingly, no species belonging to *Flavonifractor*, *Achromobacter*, or Eggerthellaceae was linked to the HC-enriched vOTUs, and several members of other genera, such as *Lawsonibacter asaccharolyticus* and *Oscillibacter* sp., were rarely connected to HC-enriched vOTUs. These findings indicate distinct virus-host relationships between AS-enriched and HC-enriched vOTUs.

### Functions of viral signatures associated with ankylosing spondylitis

**Figure 4:**
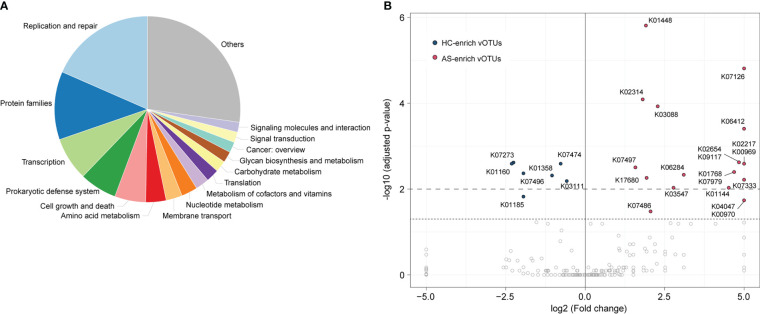
Comparison of functions between AS-enriched and HC-enriched vOTUs. **(A)** Composition of the functions of AS-associated vOTUs, as categorized at KEGG pathway level A. **(B)** Comparison of the occurrence rate of functions between AS-enriched and HC-enriched vOTUs.

To explore the functional potential of gut viral signatures associated with AS, we identified 73,576 putative protein-coding genes from 1,004 AS-associated vOTUs and annotated them against the Kyoto Encyclopedia of Genes and Genomes (KEGG) database, leading to 11,478 genes annotated with KEGG functional orthologs (KOs). These annotated genes frequently participate in replication and repair mechanisms, protein family functions, transcription, prokaryotic defense systems, cell growth and death, and amino acid metabolism, among others (**Figure 4A**). Comparison analysis revealed that the occurrence rates of 27 KOs were significantly different between HC-enriched and AS-enriched vOTUs (Fisher’s exact test, adjusted p value < 0.05; **Figure 4B**; **Table S3**). Among these KOs, 7 are encoded by HC-enriched vOTUs, including 4 enzymes involved in genetic information processing and expression (K01160, K03111, K07474, and K07496), 2 lysozymes (K01185 and K07273) and 1 caseinolytic protease (K01358). The other 20 KOs were more frequently detected in AS-enriched vOTUs, including 11 functions involving genetic information processing and expression, 5 metabolic functions, and 4 potential structure and signaling proteins. Notably, 5 metabolic functions contain K01448 (N-acetylmuramoyl-L-alanine amidase), K00969 (nicotinate-nucleotide adenylyltransferase), K02217 (ferritin), K01768 (adenylate cyclase), and K01144 (exodeoxyribonuclease V).

### Performance of the classification model based on the gut viral community

**Figure 5:**
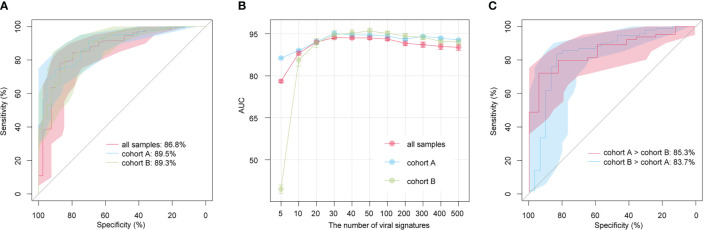
Prediction models of AS status based on gut viral signatures. **(A)** Receiver operating characteristic (ROC) analysis of the classification of disease/control status using the random forest algorithm. **(B)** Average AUC values for different numbers of AS-associated vOTUs. **(C)** ROC analysis of the classification of disease/control status in cross-cohort models.

We built classification models for the AS patients and HCs based on the relative abundances of 1,004 AS-associated vOTUs using the random forest algorithm with 5 fivefold cross-validations, and we assessed the model performance based on the AUC score. AUC scores were 86.8% (95% confidence interval [CI] = 80.1%-93.6%), 89.5% (95% CI = 84.4%-94.6%), and 89.3% (95% CI = 83.7%-94.9%) in all samples, within cohort A, and within cohort B, respectively (**Figure 5A**). Furthermore, to optimize the model, we built random forest models based on a small set of vOTUs. All AS-associated vOTUs were first ranked by the importance level (i.e., mean decrease accuracy) obtained from the model for all samples. Then, we compared the performances of the models using various numbers of top-ranked vOTUs and observed approaching the optimal performance (AUC: 93.6% to 95.1%) in the models for each dataset based on 30 top-ranked vOTUs (**Figure 5B**). Notably, the 30 top-ranked vOTUs also showed excellent potential for classifying controls vs. patients in cross-dataset prediction (AUC > 80%; **Figure 5C**). These findings suggest that gut viral signatures have high effectiveness for AS prediction.

## Discussion

AS is a chronic, progressive inflammatory disease. In addition to involving the spine, patients often have some extrajoint manifestations, such as gastrointestinal problems. Studies have found that AS is closely related to gut inflammation, and approximately 70% of AS patients have subclinical gut inflammation (33), which suggests that gut dysbiosis may contribute to this phenomenon. In recent years, many studies have shown that the occurrence of AS is influenced by the gut microbiota (1, 34). The gut microbiota mainly consists of bacteria, archaea, viruses, fungi and parasites. In a study of the terminal ileum, researchers found a higher abundance of five bacterial families (Lachnospiraceae, Prevotellaceae, Rikenellaceae, Porphyromonadaceae, and Bacteriaceae) in AS patients and a lower abundance of Rumincoccaceae and Rikenellaceae. Several bacteria play key roles in the pathogenesis of AS, such as *Klebsiella pneumoniae* and *Bacteroides vulgatus* (35). In addition, several mechanisms to explain the role of the microbiome in the etiology of AS have been proposed, such as altered intestinal permeability (36), stimulation of the immune response (37), and molecular simulation (38). However, none of these fully explain the etiology of AS, and researchers continue to search for possible etiologies.

The gut microbiota is closely related to AS and may even be one of the key inducers; however, enteroviruses are often overlooked. In this study, we explored changes in the gut viral population of 193 AS patients compared with 59 healthy controls. Our study enhances the previous metagenomic datasets based on the gut virome of AS by adding more information. Metagenome-based virome sequencing improves the observed diversity of viruses, as it can capture more information about previously overlooked viruses. Compared with the HCs, the AS patients in our study exhibited a significant decrease in viral richness (number of observed vOTUs), particularly those in cohort A, though there was no significant difference in viral evenness (Shannon index and Simpson index) between the two groups. Specifically, comparison of the family-level composition revealed that the virome AS patients had an increased abundance of *Gratiaviridae* and *Quimbyviridae* but that HCs showed a higher abundance of *Drexlerviridae* and *Schitoviridae*. *Gratiaviridae* viruses are a putative novel family of viruses that is able to infect *Bacteroides*, and it consists of less abundant viruses that are remotely related to the *Autographiviridae*, *Drexlerviridae*, and *Chaseviridae* families (39). *Quimbyviridae* members are abundant, hypervariable viruses that often infect *Bacteroides* and are suspected to be obligate lytic viruses; they are a recently described novel family of viruses that are highly abundant and widespread in the human gut (39). Enrichment of these two novel viruses in AS suggests that they may play an important role in its pathogenesis. Interestingly, many viral viruses of the *Drexlerviridae* family, such as KM18 and IME268, have cleavage activity against *Klebsiella pneumoniae* or even its biofilms, which may be due to their putative endosialidase (depolymerase) enzyme (40, 41). *Schitoviridae* is a novel viral family of *Escherichia* N4-like phages, including eight subfamilies and many new genera, and there is a poorly reported association between *Schitoviridae* and human disease (42).

We used the combined probability values from independent tests in two cohorts to obtain 1004 vOTUs with significant differences between AS patients and HCs, of which 260 were enriched in AS patients and 744 in HCs. The AS-enriched vOTUs presented a higher proportion of *Myoviridae* viruses, *Microviridae*, *p-crAss-like*, and *Gratiaviridae*; the HC-enriched vOTUs contained more viruses of *Siphoviridae*, *Drexlerviridae*, and *Podoviridae*.

From the aspect of viral host, we found that most AS-associated vOTUs could be assigned to at least one prokaryotic host, including 474 HC-enriched vOTUs and 191 AS-enriched vOTUs. The dominant hosts of AS-enriched vOTUs included members of *Flavonifractor*, *Lawsonibacter*, *Clostridium*, *Bacteroides*, *Oscillibacter*, *Achromobacter* and an unknown genus from Eggerthellaceae. HC-enriched vOTUs are predicted to frequently infect members of *Blautia, Ruminococcus*, *Collinsella*, *Prevotella*, *Faecalibacterium*, *Dorea*, *Eubacterium*, and *Bifidobacterium*. *Flavonifractor plautii* is a flavonoid-degrading bacterium that was reported to affect antigen-induced T helper 2 cell (Th2) immune responses in mice, and it is also increased in young-onset colorectal cancer patients (43, 44). There are few articles on the relationship between *Lawsonibacter* and human diseases, though one species of *Lawsonibacter* was identified as a butyrate-producing bacterium (45). Interestingly, the relative abundance of *Clostridium* was found to be decreased in the gut microbiota of AS patients in many previous reports (46–48), but we found that *Clostridium* was the dominant host of AS-enriched vOTUs. Differentially abundant taxa have been identified in patients with juvenile idiopathic arthritis with the HLA-B27 allele, including *Bilophila*, *Clostridium* cluster XIVb, *Oscillibacter*, and *Parvimonas* (49), and *Oscillibacter* was shown to be involved in the integrity of the mouse intestinal barrier (50). *Achromobacter* may be involved in the development of systemic-onset juvenile idiopathic arthritis (51). Eggobacteriaceae is considered a bacterial biomarker of alopecia universalis and radiation enteritis (52, 53). These studies suggest the close association between the host bacteria of these AS-enriched vOTUs and AS. On the other hand, the relative abundance of *Blautia* is reportedly lower in HLA-B27+ offspring (54), in accordance with our results. In addition, *Ruminococcus gnavus group*, *Faecalibacterium prausnitzii* and *Eubacterium ruminantium group* are present at a lower abundance in AS patients (48, 55). However, in contrast to our results, the relative abundances of *Collinsella*, *Prevotella, Dorea*, and *Bifidobacterium* are increased in AS patients (47, 48, 55). These findings indicate distinct virus-host relationships between AS-enriched and HC-enriched vOTUs.

We also focused on the functions of viral signatures associated with AS and found that 4 enzymes involved in genetic information processing and expression (K01160, K03111, K07474, and K07496), 2 lysozymes (K01185 and K07273) and 1 caseinolytic protease (K01358) to be encoded by HC-enriched vOTUs. Eleven functions involve genetic information processing and expression, 5 metabolic functions, and 4 potential structure and signaling proteins. Notably, 5 metabolic functions containing K01448 (N-acetylmuramoyl-L-alanine amidase), K00969 (nicotinate-nucleotide adenylyltransferase), K02217 (ferritin), K01768 (adenylate cyclase), and K01144 (exodeoxyribonuclease V) were more frequently detected in AS-enriched vOTUs. To date, many attempts to screen biomarkers of AS have been made. Based on miRNA-451a and miRNA-125a expression levels (56) and collagen protein of serum samples (57), the models generally achieved the performance of AUCs between 0.60 to 0.80. Herein, we obtained a more accurate and higher AUC of 0.868, 0.895, and 0.893 using only 1004 gut vOTUs. This analysis showed highest AUC for the model (0.936 to 0.951) when using a subset of 30 top-ranked vOTUs. These findings are encouraging developments that suggest the high diagnostic potential of the gut virome in AS discrimination.

## Conclusion

Overall, based on the cross-cohort metagenome shotgun sequencing data, we identified some viral signatures with a significant difference between AS patients and healthy controls. These viral signatures may be potential targets for treating AS, contributing to developing prevention and treatment strategies for AS from new perspectives. In addition, the excellent predictive model (AUC >0.936) using only a small number of viruses indicate its excellent potential for clinical application.

## Data availability statement

The original contributions presented in the study are included in the article/**Supplementary Material**. Further inquiries can be directed to the corresponding author.

## Ethics statement

Ethical review and approval were not required for the study on human participants in accordance with the local legislation and institutional requirements. Written informed consent for participation was not required for this study in accordance with the national legislation and the institutional requirements.

## Author contributions

CL, QY, SL, and WS conceived of the study. YanZ, SL, RG, YueZ and JMen performed data interpretation. JMa and WY performed partial data analysis. CL, YanZ, QY, CC, SL, ZW, and WS drafted the manuscript. All authors contributed to the article and approved the submitted version.
